# Status of human cystic echinococcosis based on hospital records in Mazandaran Province: A first registry-based evidence

**DOI:** 10.1016/j.parepi.2023.e00314

**Published:** 2023-07-04

**Authors:** Rabeeh Tabaripour, Ali Sharifpour, Mahdi Fakhar, Samira Asadi, Samira Esmaeili Reykandeh, Mahbobeh Montazeri, Masoud Keighobadi

**Affiliations:** a Iranian National Registry Center for Lophomoniasis and Toxoplasmosis, Imam Khomeini Hospital, Mazandaran University of Medical Sciences, Sari, Iran; b Iranian National Registry Center for Hydatid Cyst Mazandaran Branch, Imam Khomeini Hospital, Mazandaran University of Medical Sciences, Sari, Iran

**Keywords:** Human cystic echinococcosis, Hydatidosis, Hospital information system, Iran, Registry

## Abstract

**Background:**

Human cystic echinococcosis, as an emerging neglected parasitic disease, is caused by tapeworms of the genus *Echinococcus* spp. Because of the medical and economic importance, this study aims to review the epidemiology and clinical features of hydatidosis in patients admitted to medical and surgical wards in three referral teaching hospitals over 15 years in Mazandaran Province, northern Iran.

**Methods:**

Data were collected from hospital records that were accessible via the hospital information system (HIS) between 2005 and 2019 (15 years).The demographic information (age, sex, living area, and occupation), dog contact, number of the cysts, types of organs involved, and history of disease recurrence were assessed.

**Results:**

One hundred twenty-one patients with human cystic echinococcosis (CE) were involved in the study, from whom 58 patients (47.93%) were male and 63 (52.07%) were female. The majority of patients were rural residents (64.46%) and also housewife (28.10%). Based on the results, only about 16.53% of the patients had history of close contacts with dogs. The liver was the organ involved in the most cases of CE. There were statistically significant differences between residence, occupation, history of close contacts with dogs, type of organs involved, number of cysts and history of disease recurrence (*P* < 0.05).

**Conclusions:**

Our data provides valuable registry-based information about CE in an endemic region. The data highlights that most patients lived in rural areas and were housewives. Additionally, they had a low rate of disease recurrence but a high rate of close contact with dogs. Moreover, further monitoring on registry-based program and strengthening the HIS in the provincial hospitals in the studied area are required.

## 1 Introduction

Human cystic echinococcosis (CE) is an important chronic zoonotic disease in many parts of the world including Iran (Rokni, 2009; Shafiei et al., 2016; Aghajanzadeh et al., 2008), which is caused by the larval stages of the parasites belonging to the genus *Echinococcus* (McManus et al., 2003; Moro and Schantz, 2009). The adult worm lives in the intestines of canidae family (as a definitive host), livestock (as intermediate host), and humans (as an accidental host). Human affected to the larval stage of the parasite (hydatid cyst) via eating eggs excreted from dog feces (Moro and Schantz, 2009). Larvae enter the internal organs; particularly the liver, lungs, spleen, kidney, and brain, via the portal vein of the intestine, where they convert to the cystic stage (Córdoba et al., 2019). Hydatid cysts can appear in a variety of places, but the liver and lungs are the most commonly affected organs. Although symptomatic cases of the disease almost always necessitate surgery, there are several cases of asymptomatic cases that are never diagnosed until the cyst grows large enough (Islami et al., 2018). The overall annual cost of CE in Iran was estimated at US$232.3 million, including both direct and indirect costs. The cost associated with human CE was estimated at US$93.39 million and the annual cost associated with CE in livestock was estimated at US$132 million. (Fasihi Harandi et al., 2012).

Hydatid cysts have been studied clinically, in the laboratory, and epidemiologically in Iran for the past two decades, and cases have been reported from all over the country (Darani et al., 2003); (Saeed et al., 2000). In a previous systematic review, the prevalence of human CE in Iran was estimated to be 5%. The most severe cases of CE were found in the southwest and south of Iran (Shafiei et al., 2016). In another systematic review, human CE was found to be 4.2% prevalent in another systematic review, with the disease being most prevalent in rural areas and southern Iran (Khalkhali et al., 2018).

According to research, there are five *Echinococcus granulosus* (*E. granulosus*) genotypes (G1, G2, G3, G6, and G7) in Iran, and CE has affected five livestock species (sheep, goats, cattle, buffaloes, and camels). Other genotypes were found mostly in the central and western parts of Iran, with the exception of G7, which is found in Khorasan Province in eastern Iran. The following are the animal hosts for each recognized genotype in Iran: Sheep, cattle, camels, goats, and buffaloes have G1 (92.75%) and G6 (4.53%); five herbivore hosts and dogs have G3 (2.43%); dogs have G2 (0.06%); and sheep and goats have G7 (0.2%). In humans, in addition to the exclusive G1 genotype, which is dominant worldwide, G6 is the most common clade in human infections in Iran (Khademvatan et al., 2019).

In general, increased physician awareness of various clinical and diagnostic aspects of hydatid cyst will lead to earlier diagnosis of the disease and a reduction in its complications. Although similar studies on patients with hydatid cysts have been conducted in Mazandaran Province, no comprehensive study covering all operated patients in this province has been published. Therefore, with regard to the medical and economic importance of this disease, and also recently launching a provincial regional registry center for hydatid cyst in Mazandaran Province, northern Iran, we aimed to reconsider the status of CE in three referral teaching hospitals in the north of Iran.

## 2 Subjects and methods

### 2.1 Ethics approval and consent to participate

This study was reviewed and approved by the Iranian Research Ethical Committee at Mazandaran University of Medical Sciences, Sari, Iran (IR.MAZUMS.REC.1398.711). Written informed consent was obtained from the patients for publication of the Data.

### 2.2 Study area

Mazandaran Province, which covers an area of 23,842 km2, is located on the Caspian Sea's south-east coast between 36.2262° N and 52.5319° E (Otero-Abad and Torgerson, 2013). The climate is temperate, with relative humidity ranging from 70% to 100%. The annual temperature ranges between 10 and 35 °C, with rainfall ranging between 800 and 1200 mm (Mesgari et al., 2013).

### 2.3 Data collection

This retrospective study was conducted on patients admitted to the medical and surgical wards of three teaching hospitals in Mazandaran Province, northern Iran, including Imam Khomeini, Bouali Sina, and Razi Hospitals over 15 years, from 2005 to 2019. Data was collected from hospital records accessible through the hospital information system (HIS) program. The demographic information (age, sex, living area, and occupation), dog contact, number of the cysts, types of organs involved, and history of disease recurrence were evaluated.

### 2.4 Statistical analysis

Statistical analysis was conducted using SPSS software (version 23.0). The frequency of data was displayed as a percentage (%). The extended Fisher test and the Chi-square test were used to examine statistically significant differences in parametric data. A *P*-value of <0.05 was considered statistically significant.

## 3 Results

Based on collected data through the HIS program, 121 patients with CE enrolled in the study. Among them, 58 patients (47.93%) were male and 63 (52.07%) were female. The highest age of the patients was 41–60 years old, with no statistically significant difference between males and females (*P* = 0.24). The majority of patients were rural (64.46%) and housewives (28.10%). Based on the results, only about 16.53% of the patients had a history of close contact with dogs. The liver was the most commonly (61.16%) involved organ in CE cases followed by lungs (23.97%), spleen (1.65%) and brain (1.65%). There were statistically significant differences between residence, occupation, history of close contacts with dogs, type of organs involved, number of cysts, and history of disease recurrence (*P* < 0.05) (Table 1). The mean number of cysts was 1.58% ± 1.2 and most of them were single (61.16%). The results also showed the highest number (85.12%; 103/121) of registered patients were belonged to 2010–2019 years.

**Table 1:** Demographic and epidemiologic characteristics of hospitalized hydatid cyst cases in Mazandaran Province, northern Iran.

Variables	No. of patients	*P*- value

	(%)	
Gender		
Male	58 (47.93)	
Female	63 (52.07)	0.24
Age groups		
< 20	17 (14.05)	
20–40	37 (30.58)	0.11
41–60	38 (31.40)	
> 60	29 (23.97)	
Residence		
Urban	43 (35.54)	
Rural	78 (64.46)	0.01⁎
Occupation		
Herdsman	9 (7.44)	
Farmer	5 (4.13)	
Housewife	34 (28.10)	0.001⁎
Worker	3 (2.48)	
Employee	7 (5.78)	
Student	9 (7.44)	
Not specified	54 (44.63)	
History of close contacts with dogs		
Yes	20 (16.53)	0.01⁎
No	101 (83.47)	
Type of organs involved		
Liver	74 (61.16)	
Lung	26 (21.49)	
Both Liver & Lung	17 (14.05)	0.001⁎
Spleen	2 (1.65)	
Brain	2 (1.65)	
Number of cysts		
1	74 (61.16)	
2	33 (27.28)	0.01⁎
3	7 (5.78)	
>3	7 (5.78)	
History of disease recurrence		
Yes	28 (23.14)	0.02⁎
No	93 (76.86)	
Year		
2005–2009	18 (14.88)	0.11
2010–2014	51 (42.15)	
2015–2019	52 (42.97)	
Total	121	

⁎ *P* value under <0.05 means statistically significant.

## 4 Discussion

In many countries, human CE, as an emerging neglected disease, is a major public health issue that results in significant economic resource loss. This disease has a global distribution with an annual occurrence ranging from 1 to 200 per 100,000 individuals (Moro and Schantz, 2009; Fakhar et al., 2021). The prevalence of CE is different in various surveys due to factors such as climate, and infection rate in main hosts and livestock, and the level of hygiene. Furthermore, raw vegetable consumption and an increase in the number of pet owners are two other factors contributing to high prevalence in some areas (Al-Shibani et al., 2012; Kohansal et al., 2015). In Iran, the highest infection rate is reported in the western and northern parts of the country (55% in Kermanshah and 32% in Mazandaran) (Mahmoudi et al., 2019). In the present study, the hydatid cyst was detected in 121 patients during a fifteen-year period which is consistent with a 5-year analysis in Alborz Province with 26 cases, a 15-year study in Khuzestan with 360 cases, and a 10-year study in Mazandaran with 79 cases (Omidinia et al., 2020); (Kamali et al., 2018); (Islami et al., 2018). It should be noted that, because of closeness of the Mazandaran to Tehran, the capital of Iran, a large number of patients are referred to the hospitals of Tehran, for diagnostic and treatment services. Therefore, the present data will be the tip of the iceberg and did not show the real status of CE cases in the area.

According to the findings of this study, CE cases were mostly females (52.7%). This finding was similar to the results of Rezaei et al. in Qom during 2002–2013 (57.3% females) (Rezaei et al., 2014). Based on the results of Ibrahim in 2007 in Egypt, CE cases were more frequent in females (35%) than in males (Ibrahim et al., 2007). In another study in Meshkinshahr district, Ardabil Province, northwestern Iran, Heidari et al. reported that the rate of human hydatidosis is higher in males than in females (Heidari et al., 2011). In a similar study in Khuzestan and Alborz, they reported CE with a rate of 56.1% and 57.7% in females, respectively (Kamali et al., 2018); (Omidinia et al., 2020). Overall, women look like to be more exposed to infection sources (such as contaminated soil and raw vegetables) than men in some areas of Iran, particularly in rural areas (Chalechale et al., 2016; Asgari et al., 2013) and it also seems that women are more susceptible to CE than men. Moreover, the highest rate of infection was found among housewife (28.10%) and the majority of patients had no contact with dogs. On the other hand, women in the rural areas of Mazandaran Province, have a strong desire to eat raw vegetables. Consequently, they are greater probably at risk for CE in the studied area.

Based on the results of this study, most patients were in the age group of 41–60. For example, in Hamadan and Arak, the highest rates of infection were found in the 20–39 and 10–49 age categories, respectively (Asgari et al., 2013); (Ahmadi and Hamidi, 2008). The recent study found that the infection was less prevalent among children, similar to the findings of previous investigations in Yazd and Tehran (Fattahi et al., 2015); (Pezeshki et al., 2007).

The present study showed that the number (103/121) of CE registered cases were significantly increased throughout 2010–2019. The reasons for this could be the establishment of a registry center for human CE in the province and more access of patients to medical diagnostic centers and increasing the awareness of surgeons.

The results of this study showed that 78 patients (64.46%) were living in rural areas. This finding was similar to the results of cases in the provinces of Hamadan and West and East Azerbaijan (Ghabouli Mehrabani et al., 2014; Ahmadi and Hamidi, 2008; Hajipirloo et al., 2013). In rural locations, disease is more prevalent due to close contact with soil, animals, and dogs (Hajipirloo et al., 2013). Of all patients, 20 (16.53%) reported having direct contact with dogs. In another study, it was found that farmers and ranchers had the highest rate of infection (Heidari et al., 2011).

Our results showed that 74 (61.16%) and 26 (21.49%) of 121 patients had cysts in the liver and lungs, respectively. Our findings were consistent with previous research in Khuzestan and Alborz provinces (Kamali et al., 2018); (Islami et al., 2018). This result is explained by the fact that the liver and lungs are the most significant body filters and the first locations to encounter migrating parasite larvae, and a few parasites can generally escape and get access to other organs (Ahmadi and Badi, 2011).

We had no data about using immunodiagnostic techniques in this study. Antiparasitic agents, including albendazole, were used for most of the patients. Appropriate medical treatment before and after surgery may help to reduce the number of relapses (Jamshidi et al., 2008). Furthermore, in patients with many cysts, a combination of albendazole and praziquantel can be used instead of surgery (Bygott and Chiodini, 2009). The favored technique in our situation was surgical resection and open full cystectomy with the addition of a scolicidal agent.

In brief, CE must be monitored and controlled in these areas due to its importance to human health and the domestic animal sector. This necessitates public health education and awareness about the disease's dangers, as well as education on proper animal slaughtering techniques, prevention of feeding dogs by viscera of home-slaughtered animals, prevention of direct contact with dogs' feces, enforcement of meat inspection legislation, and improved veterinary services, including, most importantly, treating and vaccination of sheep and other domestic animals, and combating stray dogs (Rokni, 2009, Ziaei et al., 2011; Ziaei Hezarjaribi et al., 2017). Health education, proper disposal of infected offal's should be considered as the main control measurements to reduce the rate of CE in human and livestock in this CE-endemic area (Ziaei Hezarjaribi et al., 2017).

## 5 Conclusion

Our results showed CE is relatively common in Mazandaran Province, particularly among rural inhabitants. Thus, it still remains a major public issue in the area and also it has been increased during the last decade. Moreover, our data highlights that most patients lived in rural areas and were housewives. Additionally, they had a low rate of disease recurrence but a high rate of close contact with dogs. Further monitoring of registry-based programs and strengthening of the HIS in the provincial hospitals in the area are required. As a whole, our data provide valuable information concerning the epidemiology of CE based on registry plan in the northern region of Iran, which will likely be very significant for management and prevention plans of this disease.

## Declaration of Competing Interest

The authors declare that they have no known competing financial interests or personal relationships that could have appeared to influence the work reported in this paper.
